# Cost Assessment of Inpatient Care Episodes of Stroke in Romania

**DOI:** 10.3389/fpubh.2020.605919

**Published:** 2020-12-04

**Authors:** László Lorenzovici, Andrea Székely, Marcell Csanádi, Péter Gaál

**Affiliations:** ^1^Syreon Research Romania, Tirgu Mures, Romania; ^2^G. E. Palade University of Medicine, Pharmacy, Science and Technology, Tirgu Mures, Romania; ^3^Syreon Research Institute, Budapest, Hungary; ^4^Health Services Management Training Centre, Faculty of Health and Public Administration, Semmelweis University, Budapest, Hungary

**Keywords:** burden of disease, cost analysis, Romania, hospital costs, stroke

## Abstract

**Introduction:** Stroke is the second leading cause of death worldwide and Romania is no exception. There is a high economic burden associated with the treatment of stroke patients, which puts pressure on the healthcare budget. This study aims to measure the inpatient treatment costs of stroke patients in Romania.

**Methods:** Our retrospective analysis follows stroke patients in six Romanian hospitals at different progressivity level from different regions. Patients are identified from the official hospital databases, reported for reimbursement purposes. Mean inpatient costs incurred with the treatment of these stroke patient episodes are calculated using the gross costing method. The cost data are derived from the management control system of the study hospitals.

**Results:** 3,155 patient episodes of stroke were identified in the study hospitals. The average cost per stroke inpatient care episode sums up to EUR 995.57 (95% CI: EUR 963.74—EUR 1 027.39) in 2017, while the overall yearly healthcare burden adds up to EUR 140 million, representing 2.18% of the total national health insurance budget and a cost of EUR 7.15 per capita.

**Conclusion:** The hospital cost of stroke inpatient care episode in Romania is high and it represents a sizable part of the healthcare budget, but it is among the lowest in Europe, which can mainly be explained by the level of economic development of the country. As both the number of patients and the cost of acute care are expected to increase in the future, the economic burden of stroke is also expected to increase.

## Introduction

According to the World Health Organization, stroke was responsible for the second-most cases of death worldwide in 2018 (1). Former studies show that it is a common illness, for instance in the United States (2), and similar figures have been reported in countries of the European region. “The Burden of Stroke in Europe” study has estimated the incidence of stroke between 0.8 and 18.8%, while the incidence of the disease was estimated to be 94/100,000 in 2008, standardized to the world population (3). Placed in an international setting, the incidence, prevalence, and mortality vary greatly among the different countries and regions. The incidence of stroke (ischemic and hemorrhagic first ever stroke) in 2010 has been of 184.61 per 100,000 person-years in the USA, 115.40 in the UK, 191.22 in Germany, 116.60 in France, 340.88 in Hungary, 226.41 in Poland, and 281.47 per 100,000 person-years in Romania. The mortality in case of Romania has been reported to be 158.96 per 100,000 person-years in 2010 (4).

Treating such a high and increasing number of cases puts tremendous pressure on the healthcare budget of every country, especially lower income countries, such as Romania (5). The costs associated with the treatment of stroke patients vary greatly among different countries, even within the same income group. In Senegal the cost of treatment is USD 416 (in 1997), while in Pakistan the cost is USD 5,230 (in 1998–2001), the latter including only direct medical costs incurred during inpatient care (6). In high income countries the costs are even higher; in France, for example, the cost per patient episode (acute phase hospitalization) is estimated to be EUR 6 199.81 (in 2011) (7), while in Canada the cost sums up to USD 10544.45 (in 2008), including diagnostics, acute care services, rehabilitation, and surgical services during the inpatient stay (8). In the United States of America, the costs add up to USD 35,175 (in 2012), considering the hospital charges for the initial treatment of patients (9). The cost differences can be explained by the varying price levels and use of resources, as different countries can apply different diagnostic and treatment protocols.”

In 2017 the Romanian patients still had problems with access to intravenous thrombolysis treatment. By the end of 2019 every county had at least one stroke-ready hospital, but the thrombolysis treatment of stroke is still not as prevalent as in other EU countries (10). The annual number of intravenous thrombolysis in 2015–2016 in Romania has been of 10.3, while the average of 42 countries has been of 142 (11). The therapeutic protocol for thrombolytic and endovascular treatment in acute ischemic stroke has been developed according to the AHA/ASA (American Heart Association/American Stroke Association) guidelines and the EHRA (European Heart Rhythm Association) practical guide. According to this, once a call is made to the emergency hotline, the operator will dispatch an ambulance unit. The emergency ambulance medical staff assesses the health status of the patient, and, if they can reach an emergency care unit within 3.5 h, the patient will benefit from intravenous thrombolysis. The staff of the emergency care unit is pre-notified of the arrival of the patient in order for them to prepare the imaging equipment and a neurologist will be on call. After diagnosing the patient, the neurologist decides upon the best course of treatment. If the patient is eligible for the thrombolysis, it is administered on site, and once the intervention is performed, the patient is admitted to the neurology department of the hospital. Depending on the status of the patient and the available equipment, the patient is either treated at the neurology department or is transferred to the Intensive Care Unit (ICU). In some cases, surgical intervention is performed at the neurosurgery department, if available.

In Romania, the costs incurred with the treatment of stroke patients is financed through several channels using various payment methods (12). The acute cases admitted to hospitals receive reimbursement through DRGs (Diagnoses Related Groups) according to the specific DRG tariff of the hospital (in Romania different DRG unit fees apply for different hospitals) and the relative DRG weight of the case. In addition, there is a national stroke and ICU health program, financed by the Ministry of Health, which is meant to cover a certain part of the medication administered to the patients. The national stroke program (known as Priority actions for the interventional treatment of patients with acute stroke) is financed by the Ministry of Health and it has as its physical indicators the number of critical patients with acute stroke and the nominal accounts of treated patients; the efficiency indicators include the average financing/treated patients and the execution of the approved budget. If the patient is transferred to the ICU and the length of stay is longer than the national average, some hospitals may receive extra funding from the NHIH. Furthermore, there is a special funding from the Ministry of Health to cover (a part of the) operational costs of the ICU. Finally, hospitals receive separate funding from the NHIH to cover recent salary increases.

The Burden of Stroke study estimated the per capita spending on stroke patients around EUR 10, while non-health care costs about EUR 20, for 2015 in Romania, one of the lowest in Europe. Although the study aimed to provide an accurate cost estimate, the researchers used a top-down approach and the incomplete in-country data pose serious limitations to the findings (3).

So far, only a small number of studies were published regarding the current actual cost and detailed cost structure of stroke patients, which could explain the differences among countries and that could be used to support resource allocation decisions. The complex reimbursement schemes are not related to the actual incurred costs (13). Since pharmacoeconomic evidence is being used more and more frequently in Romania (14), it is expected that policy makers and purchasing agents will incorporate newly generated cost information, if available, into financial decision making.

The objective of this study is to assess the costs incurred during the inpatient care episode of acute stroke patients in Romanian hospitals considering all (operational) costs for a relatively large number of cases, considering the stroke costs from hospitals at various progressivity level.

## Materials and Methods

In order to assess the inpatient costs of treating stroke patients, a retrospective cost analysis was performed on 3,155 discharged cases from 6 public hospitals in Romania from different regions and different progressivity level. For cost measurement we used a specific hospital controlling software (BSoft-HIMS) and Microsoft Excel for statistical analysis. To ensure the statistical power of the sample size, Cochran's formula has been used to calculate the minimal number of cases needed for the cost measurement.

### Data Sources and Sampling Method

As of 2019, there is no stroke registry in Romania, thus no information is available on the number of patients or new cases on a yearly basis, unlike in other countries, such as Sweden, where a national registry exists since 1995 (15). Our study has identified stroke patients from the DRG performance database of the study hospitals, which is used for reimbursement purposes on a national level. This contains the primary and the secondary diagnosis of the patients, according to version 10 of the International Classification of Diseases (ICD-10-AM v. 3). Cases, which have stroke diagnosis codes (I60.0-I66.9), either as a primary or secondary diagnosis, have been included in the study.

To ensure a large sample size of patients and the representation of various inpatient care providers, several selection criteria have been taken into consideration when identifying the study hospitals: hospital category (clinical hospitals, county hospitals, city hospitals), size, complexity of the services offered, and geographic location.

In 2017, 324 hospitals reported stroke patient episodes at least once as primary diagnosis, 57 of them had more than 300 cases, which cover 81.8% of all cases reported as primary diagnosis.

Out of these 57, six public hospitals have been included in the study, treating 3,155 stroke patients in 2017, representing 3.73% of the total cases reported having stroke as primary or secondary diagnosis. Geographically these hospitals are located in 4 regions (Central Region, Southern Region, North-Western Region, and Bucharest-Ilfov Region) out of the 8 main regions of Romania. Taking into account that Romania has implemented a national unified wage system, there is no wage difference between hospitals from different regions, therefore it was not considered necessary to cover all 8 regions. After assuring that all important geographical regions are covered, hospital level was also considered, thus including in the study 2 clinical level hospitals, 2 county hospitals, and 2 city hospitals, as cots may vary at different progressivity level of hospital care.

Due to the poor-quality data available in hospital reporting and the small number of patients with thrombolysis treatment, the segmentation for thrombolysis it wasn't possible.

### Costing Methodology

Resource use and unit costs were assigned to the treated cases on the using gross costing methodology, mainly due to the fact that Romanian hospitals do not record resource use and unit costs for most inputs at a patient level (16). This method assesses the costs in a “top-down” manner, meaning that the total costs of a service are divided by the number of services performed, which results in an average cost per service (17). This costing method is often preferred for its simplicity, although it lacks sensitivity (18). Clement et al. (19) performed statistical analysis regarding the differences in costs calculated using the gross costing methodology and micro-costing, the other main type of costing methodology. Their findings did not show a specific pattern (one resulting in higher costs than the other), but they proved that there can be differences, and, in some cases, can generate outliers (19, 20).

As a general rule, patient level information is available regarding the length of stay (LoS), intensive care unit length of stay (ICU LoS), length of operation time for cases with neurosurgery interventions, and drug consumption. The other cost elements were available at department level and needed to be assigned to the cases, based on different cost drivers, such as LoS or length of operation time.

### Cost Calculation

Our study only included the direct and indirect hospital costs associated with the inpatient care of stroke patients, including the wages of medical and non-medical staff, medication costs, the cost of medical and non-medical supplies, spare parts, utility costs, and the costs of diagnostic services: laboratory, radiology, CT scans (Computerized Tomography), MRI scans (Magnetic Resonance Imaging), transfusion, sterilization, operating room, anesthesia, ICU stay, etc. Indirect costs related to hospitalization consist of administration and other overhead costs (including building maintenance, safety and security, bookkeeping, HR services, IT departments, statistics department, and other hospital level costs that cannot be associated with one specific case or patient), according to controlling methodology (21). Non-medical costs, such as relocation expenses, changes in dietary habits, changes in productivity, social costs, etc. are not included in the study.

Resource utilization data has been extracted from the hospital medical databases, along with their internal records, registries and their management control systems. Cost elements have been extracted from the management control systems of the hospitals (which heavily rely on the balance sheet and general ledger). All cost data have been recorded in Romanian Leu (RON) and for the conversion the yearly average exchange rate was used, as calculated by the Romanian National Bank for 2017 (1 EUR = 4.56 RON).

Inpatient care is considered from the time of the patients' admission into the hospital until the time of discharge, defined as one episode of care. Hospital readmissions were treated as a new inpatient episode, independent of the previous hospitalization.

Calculations are based on hospital perspective, regardless of the reimbursement or the income of the hospitals for a patient episode. As it has been pointed out before, hospitals are reimbursed through several different channels and payment mechanisms, including DRGs, chronic financing (per diem), national health programs, subventions for salaries, etc.

**Figure 1:**
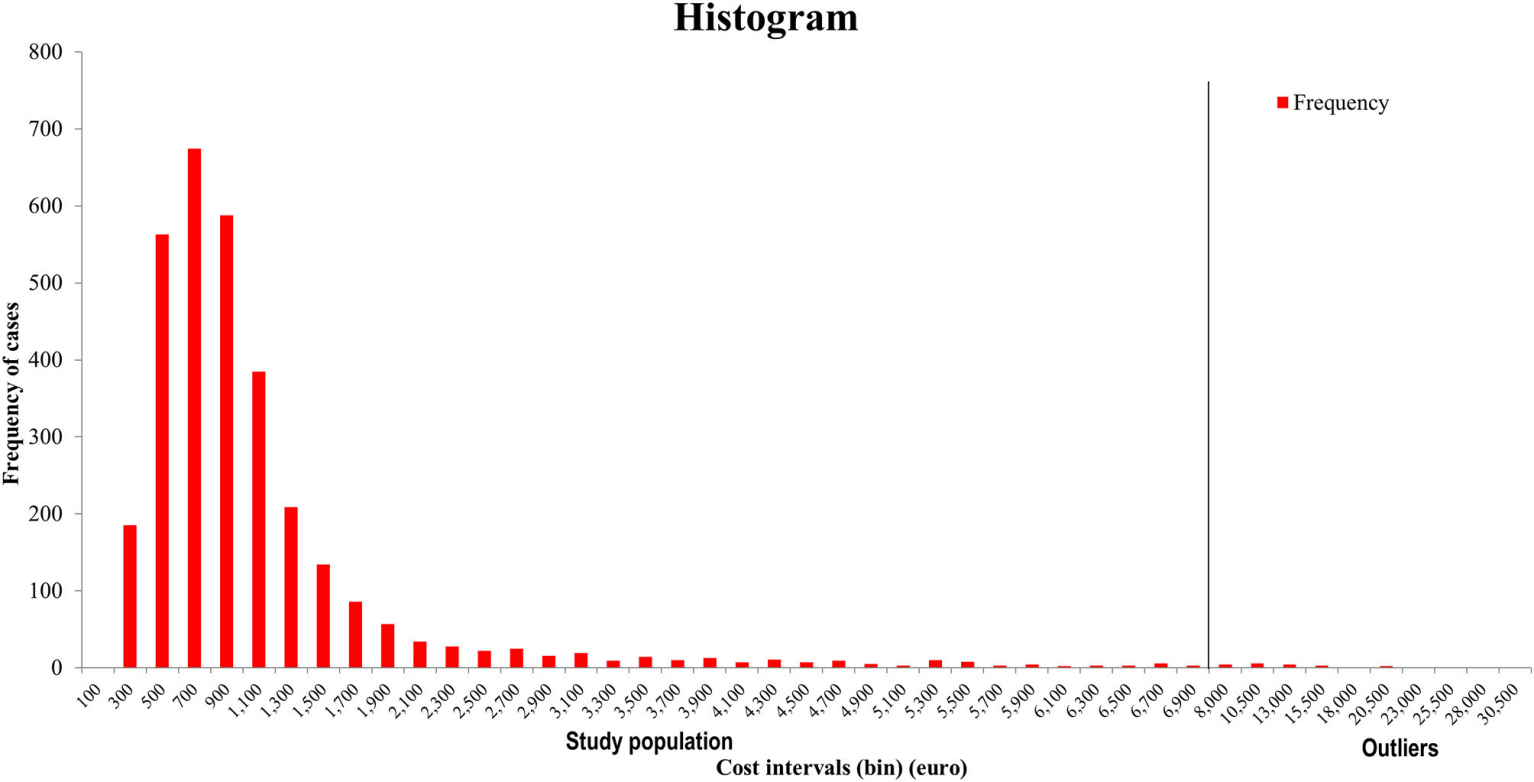
The distribution of cost of cases.

Cases that had an associated cost exceeding EUR 7500, where the continuity of the distribution brakes (Figure 1), have been considered outliers. In such cases the cost has been driven up by the exceptionally lengthy ICU stay (i.e., coma). The number of outliers was low, only 22 cases (0.69%), which were excluded from the analysis.

Amortization (depreciation) costs were not included in the cost calculations.

## Results

### Patient Population

Based on the ICD-10 diagnosis codes, at a national level in 2017, 140,719 cases (national hospital reporting database) have been reported by the hospitals to the NHIH for reimbursement (note that this is not the number of stroke patients, but inpatient episodes), most of the cases (66.4%) having been treated at the neurology department of hospitals. 9.3% of the cases were treated at the internal medicine ward (which also includes neurology in smaller hospitals), 5.6% at cardiology, 3.1% at neurosurgery, 2.1% at Urology, 1.7% at cardiology, and 1.3% at neurological rehabilitation department, while the remaining 10.5% was treated at other departments.

In our study, a total number of 3 155 cases with stroke were included, representing 2.25% of the total number of Romanian stroke inpatient episodes of 2017, after excluding the outliers, representing all the reported stroke cases in the analyzed time-frame by these 6 hospitals from the selected departments. There was a total of 22 cases (outliers) eliminated from 4 hospitals, with 8, 8, 5, and 1 case per hospital. Table 1 shows the distribution by departments of the studied cases.

**Table 1:** Number of patients per hospital department included in cost study.

**Hospital/department**	**Neurology**	**Cardiology**	**Neurosurg**.	**Internal medicine**	**Neurol. Rehab**.	**Balneo Rehab**.	**Palliative care**	**Total case**
Clinical H. 1	0	78	145	0	83	0	0	306
Clinical H. 2	160	97	85	72	0	0	0	414
County H. 1	662	10	0	5	0	0	0	677
County H. 2	1,415	32	0	52	15	26	12	1,552
City H. 1	105	12	0	4	0	0	0	121
City H. 2	81	2	0	0	0	2	0	85
Total case	2,423	231	230	133	98	28	12	3,155
Total %	76.8%	7.3%	7.3%	4.2%	3.1%	0.9%	0.4%	100.0%

The needed minimal number of cases for the results to be statistically relevant was calculated to be 207 (*SD* = 0.66, *SE* = 0.1) for the calculated average cost to have a ±10% precision in 95% of the cases (Cochran's formula), which was well exceeded by the sample size of the study.

The included inpatient episodes have been treated at the neurology, cardiology, neurosurgery, palliative care, neurological rehabilitation, and balneotherapy rehabilitation departments of the study hospitals. Table 2 shows the characteristics of the included inpatient episodes.

**Table 2:** Patient episode characteristics.

**Factor**	**Male (95% CI)**	**Female (95% CI)**	**Total (95% CI)**
	**(*n* = 1,642)**	**(*n* = 1,513)**	**(*n* = 3,155)**
Age (years)	68 (67.55–68.45)	72.48 (72.04–72.93)	70.15 (69.7–70.61)
ALoS (days)	10.02 (9.73–10.31)	9.94 (9.68–10.21)	9.98 (9.71–10.26)
ALoS ICU (days)	0.35 (0.29–0.42)	0.43 (0.36–0.5)	0.39 (0.32–0.46)
Operation time (hours)	0.07 (0.06–0.09)	0.04 (0.03–0.05)	0.06 (0.04–0.07)

*ALoS, average length of stay; ALoS ICU, average length of stay at the intensive care unit*.

52% of all inpatient episodes included in the cost assessment were male, with the average age of 68.0 for male and 72.5 for female patients. There were no major differences in ALoS (Average Length of Stay) between male and female patients, 10.02 and 9.94, respectively. Stroke patients did not spend lengthy periods of time at the ICU, with an average of 0.35 days for male patients and 0.43 days for female patients, 19.1% of male and 19.7% of female patients were treated at the ICU. Few patients underwent surgery at the neurosurgery department, with an average of 0.07 h for male and 0.04 h for female patients, 3.5% of male and 2.0% of female patients had surgery.

### Cost Data

All direct and indirect (overhead, etc.) cost items have been allocated to the treated stroke inpatient episodes on the basis of resource use data and the associated unit cost. As a result, the average cost per inpatient episode sums up to EUR 995.57. Different categories of hospitals had different average costs per inpatient episode: EUR 897.10 for the small city hospitals, EUR 813.00 for county hospitals, and EUR 1 588.93 for clinical hospitals. The list of average total cost per inpatient episode for each study hospital can be found in Table 3. Table 4 presents the average cost per different cost items, Table 5 presents the cost of medication per inpatient episode as an absolute value and as the proportion of the total average cost, while Table 6 presents the cost categories for each hospital.

**Table 3:** Average total cost/patient episode (in EUR) for each hospital included in the study.

**Hospital type/region**	**Average total cost/patient episode (€)**	**(95% CI)**
	**995.57**	**(963.74–1,027.39)**
Clinical hospital 1 (Bucharest-Ilfov R.)	2,074.18	(2,015.64–2,132.72)
Clinical hospital 2 (Central R.)	1,230.27	(1,185.86–1,274.68)
County hospital 1 (Southern R.)	903.39	(886.71–920.08)
County hospital 2 (North-Western R.)	773.57	(755.2–791.93)
City Hospital 1 (Central R.)	816.70	(807.55–825.84)
City Hospital 2 (Central R.)	1,011.55	(987.65–1,035.44)

**Table 4:** Average stroke inpatient episode cost by cost items (in EUR).

**Factor**	**Value**	**(95% CI)**
Average total cost/patient episode	995.57	(963.74–1,027.39)
Hospital ward costs	634.19	(612.61–655.77)
ICU costs	59.20	(48.42–69.98)
Medication costs	70.87	(67.35–74.39)
Operating room costs	11.38	(8.44–14.32)
Emergency care service costs	44.65	(43.85–45.45)
Diagnostic services costs	98.81	(93.93–103.70)
Other costs	16.87	(16.19–17.56)
Overhead costs	59.59	(58.09–61.09)

**Table 5:** Average cost of medication per stroke inpatient episode (in EUR).

**Hospital type**	**% of medication cost from total cost**	**Medication cost/case**
Clinical hospital 1	5.99%	124.22
Clinical hospital 2	7.63%	93.88
County hospital 1	9.83%	88.80
County hospital 2	6.49%	50.24
City Hospital 1	6.75%	55.15
City Hospital 2	2.27%	23.01

**Table 6:** Average stroke inpatient episode cost by hospital and by cost items (in EUR).

**Hospital/cost item (€)**	**Total cost**	**Hospit. ward**	**ICU costs**	**Medication**	**Operating R**.	**Emergency c**.	**Diagnostic serv**.	**Other costs**	**Overhead**
Clinical H. 1	2,074.2	1,333.0	116.4	124.2	103.8	43.4	253.8	21.2	78.4
Clinical H. 2	1,230.3	638.9	244.9	93.9	10.0	20.4	150.2	11.9	60.2
County H. 1	903.4	561.4	13.3	88.8	0.0	62.9	94.1	20.1	62.9
County H. 2	773.6	527.0	20.6	50.2	0.0	46.8	59.1	16.0	53.8
City H. 1	816.7	585.2	0.0	55.2	0.0	9.4	71.3	21.4	74.3
City H. 2	1,011.6	702.4	104.4	23.0	0.0	32.4	91.9	10.2	47.3

**Figure 2:**
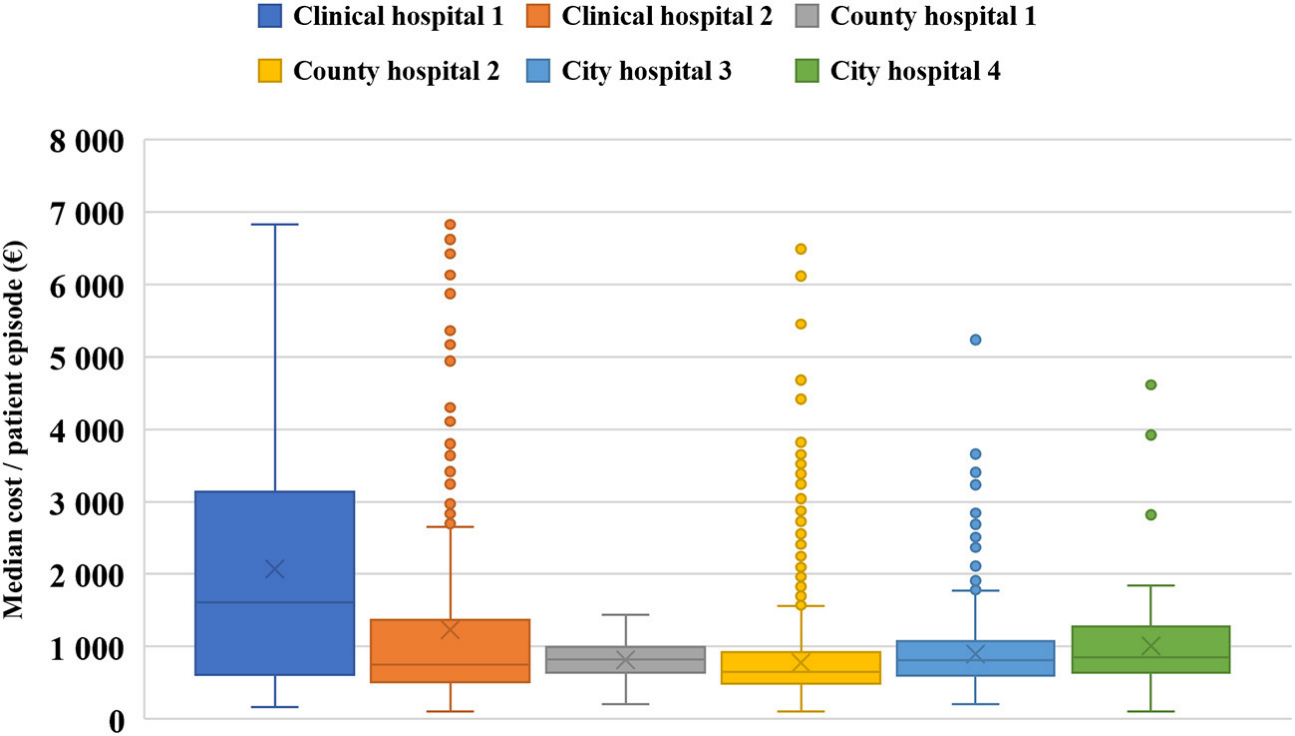
Median (—) and Average (X) total cost/patient episode (in EUR) for each hospital included in the study.

Figure 2 shows the average, median, and distribution of the costs/patient for the 6 hospitals included in the study.

Sixty-nine percent of the EUR 995.57 average cost are attributed to the care and treatment received by the patients at the hospital ward, 7.73% are attributed to ICU care costs, and 1.14% to operating room costs. Costs at the emergency care unit represent 4.49% of the total average cost. Diagnostic services are also a notable cost item with close to one tenth (9.93%) of the total average cost. The remaining costs consist of the overhead (mainly administrative) costs at 5.99% and some other costs at 1.70%.

Our study shows that the highest cost associated with the treatment of stroke inpatient episode can be attributed to the hospital ward stay, mainly due to the prolonged LoS of almost 10 days per patient episode. Given the typical course of the illness, it is not surprising that the second largest cost item is the cost of diagnostics, consisting of laboratory tests and imaging diagnostics, mostly CT and MRI scans. Although, the most expensive cost item in the inpatient care setting, in general, is the ICU cost and operating room costs, these cost items represent a smaller share of the total cost of stroke episodes, we observed that stroke patients spend little time at the ICU (9.6% of the patients with an average length of stay at the ICU of 4.15 days) and only 4.2% of the patients undergo surgery, with an average operating time of 1.34 hours per patient.

**Figure 3:**
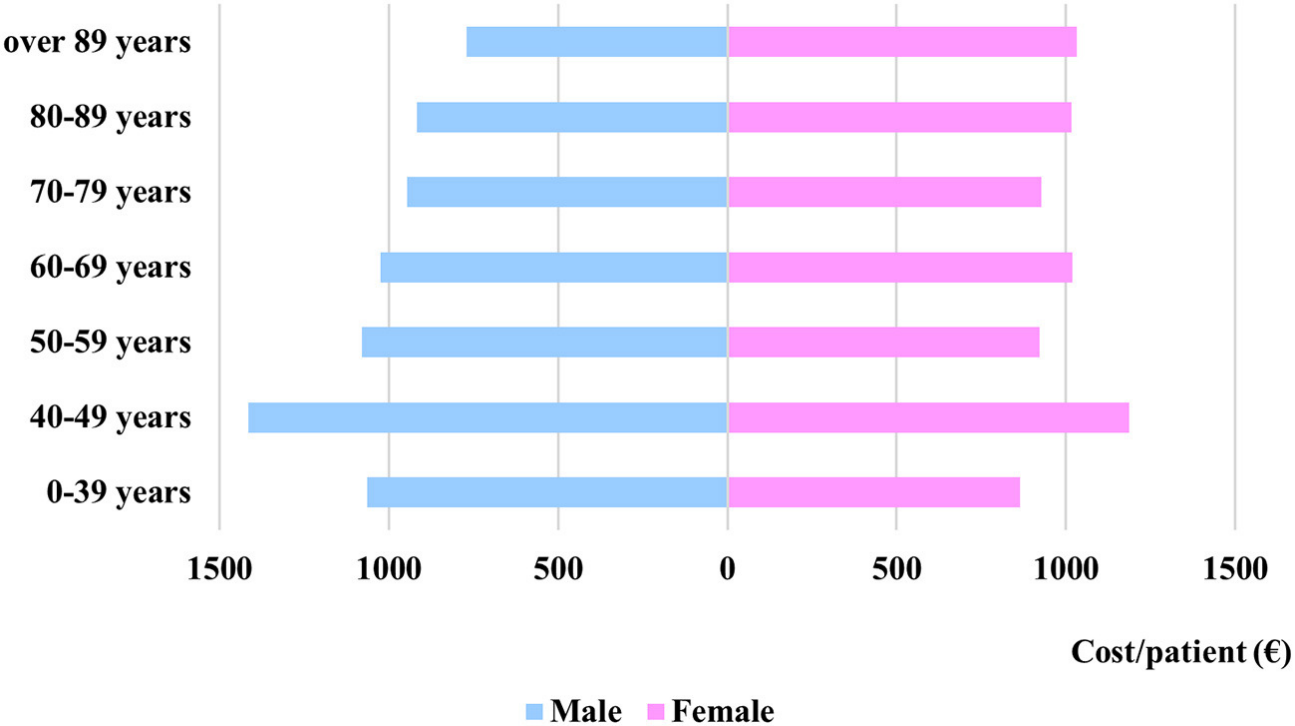
Average cost of stroke patients per age category and sex.

**Figure 4:**
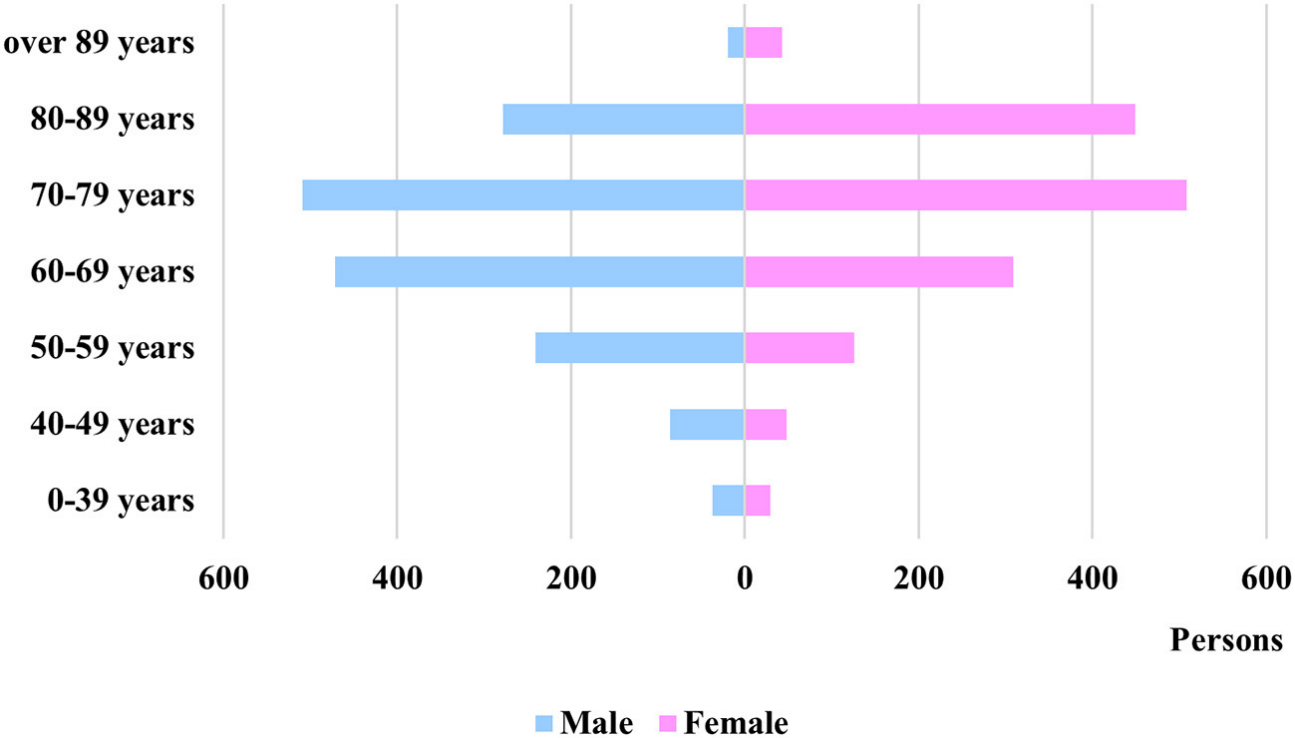
Distribution of patients based on sex.

Regarding the cost per age category and gender, some variance can be observed, as it can be seen in Figure 3, while the patient distribution between sexes can be seen in Figure 4.

The average costs per patient episode of male and female patients show no statistical difference (male = EUR 975.96, female = EUR 981.65, *p* = 0.41).

### Readmission Phenomenon

174 cases, representing 5.5% of all studied patient episodes have been readmissions in the same hospital in the studied period (12 months), with patients having a previous admission with stroke diagnosis (as primary or secondary diagnosis). The costs related to the admission type is presented in Table 7.

**Table 7:** Average stroke inpatient episode cost by admission (in EUR).

**Admission**	**Value**	**(95% CI)**
First admission	990.48	(958.86–1,022.1)
Readmission	1,082.15	(1,047.04–1,117.27)

The costs of the first admission and readmission show no statistical difference (*p* = 0.19).

### Budget Impact (Burden of Disease)

Considering the total number of 140,719 inpatient episodes per year and the average cost per patient episode of EUR 995.57, the total yearly budget impact sums up to over EUR 140 million, which represented 2.18% of the total 2017 NHIH budget of Romania. Nevertheless, this cost estimate is only a part of the total cost of the disease, because it covers only the inpatient costs incurred. In order to get a more complete cost estimate, the productivity losses due to deaths and disability, along with the costs of care and rehabilitation of the disabled population should also be taken into account.

## Discussion

Despite Romania being among the least costly countries of Europe in terms of inpatient care of stroke patients, our study confirms that the economic burden of treating stroke episodes puts considerable pressure on the scarce resources of the Romanian healthcare system.

Our results regarding cost of stroke inpatient episode being EUR 995.57 in 2017 (with low variability in the costs, the frequency of cases with a given total cost can be found in Figure 1) are comparable with another similar Romanian stroke cost measuring study, made in one clinical hospital (10), but are very different from the estimates of the “The Burden of Stroke in Europe” study (3), which is not surprising, given the different cost assessment methods, and the limitations of data availability. Our estimate puts Romania at the bottom of the EU countries' list of healthcare costs of stroke inpatient episodes; there are few countries with costs so low, even though there has been a substantial wage increase in Romania for health workers to decrease the gap between the wages of Romania and Western Europe. To our knowledge, no other study has been carried out to assess the costs associated with the treatment of stroke patients in Romania in many hospitals from different regions and at different progressivity level, and cost assessments for other illnesses are also close to none.

Although numerous cost measurement studies have been published in this field from other countries, it is difficult to compare the results due to the variation in the follow-up time. Some studies follow the patients for the first year, while others for longer periods of time. Aside from this, there are few studies available from Eastern Europe, which would be truly comparable with the Romanian results (22). The GDP (gross domestic product)/capita of a country could be related to the costs measured (Table 8).

**Table 8:** GDP/capita in EUR, 2017 (23–25).

**Country**	**GDP/Capita (€)**	**Mean cost of stroke (in PPP)**
Romania	9,600	996
Poland	12,200	1,238
Hungary	12,700	628
Spain	25,100	1,932
Switzerland	27,930	5,905
Italy	28,500	4,586
France	34,300	4,337
England	35,400	6,122
Germany	39,600	4,357
Netherlands	43,000	6,873
Denmark	50,800	3,362

Hospitals in different categories exhibit a notable variation in costs, which is associated with their area of attraction, professional level, corresponding medical staff, and infrastructure (21). For instance, university (clinical) hospitals are better equipped with cutting-edge technology and are able to attend to more complicated cases as opposed to city hospitals. On the other hand, county hospitals can achieve a higher patient turnover, while city hospitals struggle to achieve economies of scale. Another influencing factor on the cost level is the neurosurgical services offered by higher progressivity level hospitals for stroke cases, if necessary.

Regarding the cost structure of inpatient episodes, the findings of our study are in line with the results of other studies. Diringer et al. (26), for instance, has found room charges to be around 50% of the total costs in the USA, with the notable difference in resource use, as the average length of stay of 10 days in Romania is higher than the 6 days reported from the USA. This explains most of variation of costs between countries. Similar results were reported from Singapore, where the ward costs represented 48% of the total costs (27), while other studies have found this to be between 43 and 83% (28).

Analyzing the readmission phenomenon, our findings are comparable with other study performed in Romania in one hospital (~11–14%), but for 2 years period (10).

The study presenting the healthcare costs of stroke patients in Switzerland shows that hospitalization is the third largest cost component (21% of all costs), after the costs of rehabilitation clinic and nursing home (23). However, the same might not apply to Romania, mostly because of the lower wages of the healthcare system and the general lower price levels. Also, these findings are not in line with the results of studies from other European countries. Inpatient costs represent about 70–73% of all costs associated with stroke care in France, Germany, Sweden, and the UK (29). These results seem more applicable for the realities of the Romanian healthcare system.

Putting the total budget impact of stroke care into perspective, GBP 911.48 million (EUR 1 104.38 million) was reported for the UK in 2007 (30). Our estimate for Romania (EUR 140 million) is about one-eighth of this figure. Although there is little to no information regarding the cost of stroke care in Eastern Europe, there is information available regarding the health insurance expenditures on stroke in Hungary, which was HUF 30.667 billion (or EUR 111.35 million) in 2010 (31). These differences cannot be explained by variation in health care need. Adjusting the data for population size, which is the crudest proxy for healthcare needs, per capita costs in the UK (with a population of 59.9 in 2007) was EUR 18.45, in Romania (with a population of 19.6 million in 2017) it was EUR 7.15, while in Hungary (with a population of 9.8 million in 2010) it was EUR 11.42. All these suggest that the total spending on stroke has a much stronger association with the wealth of the country than healthcare needs.

### Limitations

The database for reimbursement only contains inpatient episodes, thus the actual number of patients affected by stroke could be different than the number of cases reported, mainly because of readmissions and the patients deceased even before reaching the hospital.

Cost information is limited in the Romanian healthcare system. The current study only considers the cost of inpatient care of stroke patients. The capital costs of hospitals and other healthcare costs are not taken into account, such as GP visits, ambulatory care, cost of subsidized medication, rehabilitation costs, and the cost of transportation to the healthcare providers. Societal costs should also be taken into account when calculating the total cost of stroke patients, including the cost of making changes in one's living arrangements, eating habits, etc. The study does not consider any societal indirect costs (productivity losses), such as temporary work incapacity (sick leave), permanent work incapacity (disability), or absenteeism as a result of the illness. Although these costs are smaller than those in Western Europe or the US, if considering the total societal cost of stroke patients, these should also be taken into account.

There is some difficulty in comparing the results of various studies, as the included cost elements (direct and indirect inpatient costs) might differ from one study to the other along with the considered time horizon. However, this can be overcome by comparing partial costs, as the majority of studies report results per inpatient episodes.

One of the greatest challenges is that there is not much patient level information, so there is need for a gross costing approach, based on the total resource use of a given category and then allocating these to the patient level.

Our study does not differentiate the various types of stroke (ischemic stroke, hemorrhagic stroke, etc.), but calculates an average cost for all the cases treated in hospitals. Other studies show significant differences in costs when treating various types of stroke (32).

Calculating the total budget impact based on data from a limited number of hospitals also brings some level of uncertainty, especially because of the differences between the average costs of different hospitals.

Of the 8 geographic regions of the country, half have been represented in the study, thus the results carry some level of uncertainty. However, due to the universal wage system across all personnel in the public healthcare system and the fact that similar public hospitals are financed at the same level all across the country, major differences are not expected due to the geographic location of the hospitals.

In spite of these limitations, due to the high number of cases included in the study from hospitals of different progressivity levels, we consider that it provides strong evidence regarding the cost of stroke and its burden on the Romanian health care system.

## Conclusions

The costs associated with the inpatient care of stroke patients in Romanian hospitals is higher than assessed by former studies with simpler calculation methods, but still lags far behind the costs reported even by other lower- and middle-income countries. The differences among countries are best explained by their wealth and not the variation in healthcare needs, which is in line with the findings of international comparative studies on health expenditures as a whole. Nevertheless, as the number of cases is expected to increase in the near future, the economic burden of stroke patients will only put more pressure on the limited resources of the Romanian healthcare system and on the economy as a whole, which cannot be effectively addressed without analyzing the costs of the illness as the basis of decision-making and appropriate intervention in terms of the organization and financing of health services. The findings of our study can be used in cost-effectiveness analyses, when analyzing the impact of different treatment options on cost drivers. By knowing the number of stroke cases, the cost and the cost structure of care, combined with data on clinical effectiveness, more informed budget allocation decisions can be made at a systematic level.

## Data Availability Statement

The raw data supporting the conclusions of this article will be made available by the authors, without undue reservation.

## Author Contributions

LL and AS contributed to the concept and design of the study and the cost analysis. PG offered support on health policy framework. MC offered support on statistical analysis. All authors contributed to manuscript revision, read, and approved the submitted version.

## Conflict of Interest

LL and AS were employed by the company Syreon Research Romania received financial support from RNPPA. The remaining authors declare that the research was conducted in the absence of any commercial or financial relationships that could be construed as a potential conflict of interest.

## Abbreviations

AHA/ASA: American Heart Association/American Stroke Association
EHRA: European Heart Rhythm Association
ICU: intensive care unit
DRG: diagnoses related groups
H.: hospital
NHIH: National Health Insurance House
ICD: International Classification of Diseases
LoS: length of stay
ALoS: average length of stay
CT: computerized tomography
MRI: magnetic resonance imaging
GDP: gross domestic product.
